# A 10-year retrospective study of lung cancer in Uganda

**DOI:** 10.1186/s12885-022-09300-1

**Published:** 2022-02-23

**Authors:** Naghib Bogere, Felix Bongomin, Andrew Katende, Blair Andrew Omaido, Elizabeth Namukwaya, Harriet Mayanja-Kizza, Victoria Walusansa

**Affiliations:** 1 Uganda Cancer Institute, P. O. Box 3935, Kampala, Uganda; 2 Department of Medicine, Habib Medical School, Islamic University in Uganda, Kampala, Uganda; 3 Department of Medical Microbiology & Immunology, Faculty of Medicine, Gulu University, Gulu, Uganda; 4 Ifakara Health Institute, Morogoro, Tanzania; 5 Clinical Epidemiology Unit, College of Health Sciences, Makerere University, Kampala, Uganda; 6 Department of Medicine, College of Health Sciences, Makerere University, Kampala, Uganda

**Keywords:** Lung cancer, Survival, Adenocarcinoma, Uganda

## Abstract

**Background:**

Lung cancer is a leading cause of cancer-related deaths in Uganda. In this study, we aimed to describe the baseline characteristics and survival of patients with lung cancer at the Uganda Cancer Institute (UCI).

**Methods:**

We retrospectively reviewed medical records of all patients with a histological diagnosis of lung cancer registered at UCI between January 2008 and August 2018. Data on demographic, clinical, and treatment characteristics, and vital status were abstracted and analyzed. Patients with undocumented vital status on the medical records were contacted through phone calls. We determined survival as time from histological diagnosis to death. The Kaplan-Meier survival analysis was performed to estimate the median survival time and the 5-year overall survival rate.

**Results:**

Of the 207 patients enrolled, 56.5% (*n* = 117) were female, median age was 60 years (range: 20–94), 78.7% (*n* = 163) were never-smokers and 18 (8.7%) were living with HIV. Presumptive anti-tuberculosis treatment was given to 23.2% (*n* = 48). Majority had non-small cell lung cancer (96.6%, *n* = 200) with 74.5% (*n* = 149) adenocarcinoma and 19% (*n* = 38) squamous cell carcinoma. All had advanced (stage III or IV) disease with 96.1% (*n* = 199) in stage IV. Chemotherapy (44.9%, *n* = 93) and biological therapy (34.8%, *n* = 72) were the commonest treatments used. Overall survival at 6 months, 1-, 2- and 5-years was 41.7, 29.7, 11.8, and 1.7%, respectively. The median survival time of 4.4 months was not statistically significantly different between participants with NSCLC or SCLC (4.5 versus 3.9 months, *p = .335*).

**Conclusion:**

In Uganda, adenocarcinoma is the predominant histologic subtype of lung cancer and patients are predominantly females, and non-smokers. Patients present late with advanced disease and poor overall survival. Public awareness should be heightened to facilitate early detection and improve outcomes.

## Introduction

Lung cancer is the most cancer worldwide, with most cases diagnosed in less developed countries [1, 2]. In these countries, essential information guiding action on prevention and treatment of lung cancer is either unavailable or of insufficient quality at best.

In Uganda, lung cancer is the 13th most common cancer and its incidence is increasing which threatens to exert heavy morbidity, mortality, and economic cost to the country [3, 4]. Significant differences exist in survival between patients with lung cancer in Uganda and those in the western world, with survival estimates in Uganda being worse than those in the western world despite having a seemingly similar disease [3, 5]. However, vital statistics on lung cancer in Uganda are sparse and most of the current management guidelines are extrapolated from the western world. Therefore, in this study, we set out to describe the characteristics of patients with lung cancer and their survival.

## Methods

### Study setting

Uganda Cancer Institute (UCI) is a 120-bed, national cancer referral center, located in Kampala and serves most cancer patients in Uganda.

### Study design and data collection

We conducted a retrospective chart review of patients with a histologically confirmed diagnosis of lung cancer at UCI between 1st January 2008 and 31st August 2018. The following data were obtained from the medical records: Patient factors: age, sex, body mass index, presenting complaints, comorbidities, history of smoking, weight loss, vital status (dead or alive or lost to follow up); disease factors: histology, immunohistochemistry, stage, sites of metastasis, malignant pleural effusion, superior vena cava obstruction; treatment factors: modalities of treatment (radiotherapy, chemotherapy or surgery), bisphosphonate use, type of chemotherapy used during first-line treatment, and the number of cycles of chemotherapy received.

### Data analysis

Categorical variables were summarized as frequencies and percentages, continuous variables were presented as means SD, or medians with interquartile ranges. Comparison of baseline characteristics between female and male patients was performed using a Mann–Whitney U test for continuous variables and Fisher exact test for categorical data. All *p*-values were two-tailed, with statistical significance set at *p* < 0.05. Prognostic factors were analyzed using the Cox proportional hazards model, and Kaplan-Meier survival analysis was used to evaluate differences in survival rates. Statistical analyses were performed using STATA version 16.0 for Windows.

## Results

### Baseline characteristics

We enrolled 207 lung cancer patients and their baseline characteristics are summarized in Table 1**.** Majority were female (56.5%, *n* = 117), median age was 60 (20–94) years, 78.7% (*n* = 163) were never-smokers and 8.7% (*n* = 18) were living with HIV. Fourteen (6.8%) had diabetes mellitus, 4 (1.9%) had asthma and 2 (1.0%) had chronic kidney disease. Patients with HIV were more likely to be diagnosed with lung cancer below the age of 60 than HIV-negative patients (94.0% versus 42.6% respectively; *p < 0.001*).

**Table 1:** Baseline characteristics of lung cancer patients

Variables	Female (***n*** = 117) Freq (%)	Male (***n*** = 90) Freq (%)	Total (***n*** = 207) Freq (%)	***P-value***
Age in years, median (IQR)	60 (30–89)	58.5 (20–94)	60 (20–94)	*.579*
Ever smoker Unknown	9 (7.7) 2 (1.7)	35 (38.9) 0 (0.0)	44 (21.3) 2 (1.0)	.0001
Smoking duration in pack-years, median (IQR)	8.5 (7–10)	16 (1–50)	15(1–50)	.568
Alcohol use Unknown	29 (24.7) 2 (1.7)	50 (55.6) 1 (1.1)	79 (38.2) 3 (1.4)	.0001
Family history of cancer Unknown	17 (14.5) 5 (4.3)	13 (14.4) 4 (4.4)	30 (14.5) 9 (4.3)	.999
HIV Positive Unknown	11 (9.4) 1 (0.9)	7 (7.8) 0 (0.0)	18 (8.7) 1 (0.5)	*.805*
**ECOG score**				.935
0 1 2 3 4 Unknown	5 (4.3) 37 (31.6) 39 (33.3) 22 (18.8) 7 (6.0) 7 (6.0)	4 (4.4) 30 (33.3) 36 (40.0) 15 (16.7) 4 (4.4) 1 (1.1)	9 (4.3) 67 (32.4) 75 (36.2) 37 (17.9) 11 (5.3) 8 (3.9)	
BMI, median (IQR)	23.9 (14.2–43.6)	20.7 (14.5–32.9)	21.7(14.2–43.6)	.016
**Lung cancer type**				
Adenocarcinoma Squamous cell carcinoma Other NSCLC SCLC	83 (70.9) 22 (18.8) 9 (7.7) 3 (2.6)	66 (73.3) 16 (17.8) 4 (4.4) 4 (4.4)	149 (72.0) 38 (18.4) 13 (6.3) 7 (3.4)	
**Stage**				.966
IIIB IIIC IVA IVB Unknown	2 (1.7) 2 (1.7) 59 (50.4) 54 (46.2) 0 (0.0)	1 (1.1) 2 (2.2) 43 (47.8) 43 (47.8) 1 (1.1)	3 (1.4) 4 (1.9) 102 (49.3) 90 (43.5) 1 (0.5)	

Abbreviations: *BMI* body mass index, *ECOG* Eastern Cooperative Oncology Group, *IQR* Interquartile range, *NSCLC* Non-Small Cell Lung Cancer, *SCLC* Small Cell Lung Cancer

**Fig. 1:**
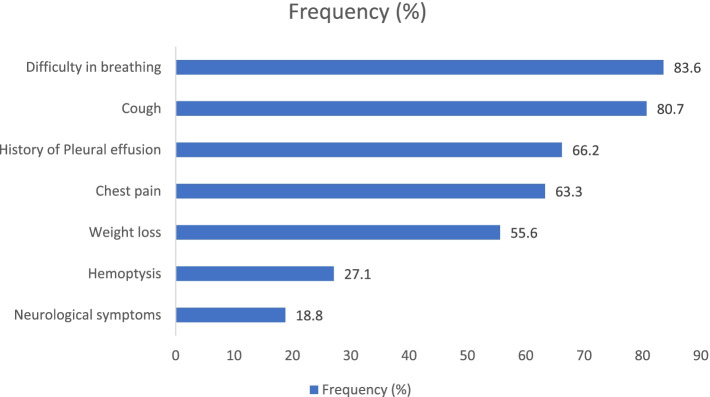
Patient presenting complaints

The presenting complaints are summarized in Fig. 1. Forty-eight (23.2%) patients were presumptively treated for pulmonary tuberculosis (PTB) for a median duration of 12 weeks (2–32 weeks) before a diagnosis of lung cancer was made. These patients had a long duration from first symptom to presentation at UCI compared to those who did not receive presumptive TB treatment (40 weeks versus 24 weeks, *p* = 0.012). A significantly higher proportion (8 of 18 patients, 44%) of HIV-positive patients were presumptively treated for PTB before a lung cancer diagnosis was made compared to those who were HIV negative (39 of 182 patients, 21.4%) *(p = 0.040).* The median duration since first symptom was 6 months (0.5–70 months). The median duration since first symptom was longer among HIV positive than HIV negative patients but the difference was not statistically significant (40 versus 24 weeks respectively, *p = .347*).

Majority of patients with history of smoking were male (80%, *n* = 35) while majority of patients exposed to passive smoke were female (81.8%, *n* = 11). Two (1.0%) of 207 patients had a history of chewing tobacco leaves. Two patients had a history of prior malignancy and had had cervical cancer and had received radiotherapy to the pelvis. Other observed risk factors included; sleeping in asbestos roofed houses (3 patients), exposure to pesticides (2 patients), exposure to industrial chemicals like photography chemicals, printer chemicals, and paints (3 patients). Biomass exposure (e.g., firewood) was only documented for 1 patient.

### Disease characteristics

Overall, 199 (96.1%) of 207 patients of patients presented with metastatic lung cancer. All the patients with SCLC had extensive stage at diagnosis. Tumor differentiation was only described for 81 patients who all had NSCLC. Of these 81 patients, 11 (13.6%) were well-differentiated, 43 (53.1%) were moderately differentiated, and 27 (33.3%) were poorly differentiated.

Immunohistochemistry was done for 18 patients who all had NSCLC. Of these, 16 were CK7 positive and 12 were TTF1 positive. EGFR mutation status was done for 6 patients and 1 was found positive. A higher proportion of patients with NSCLC had pleural metastases compared to those with SCLC (28.6% versus 78%; *p = 0.004)*. The disease characteristics are summarized in Table 2.

**Table 2:** Disease factors according to lung cancer type

Variable	Female (***n*** = 117)	Male (***n*** = 90)	Overall (***N*** = 207)	***P-value***
**Stage**				n/a
IIIB IIIC IVA IVB Limited Extensive Unknown	2 2 59 51 0 (0.0) 3 (100.0) 0 (0.0)	1 (1.5) 2 (2.0) 43 (51.0) 39 (45.0) 0 4 1 (0.5)	3 (1.4) 4 (1.9) 102 (49.3) 90 (43.5) 0 (0.0) 7 (3.4) 1 (0.5)	
**Histologic type**				.471
NSCLC SCLC	114 3	86 4	200 7	
**Liver metastases** Unknown	12 (14.3) 15 (0.0)	18 (14.5) 9 (12.0)	30 (14.5) 24 (11.6)	.071
**Contralateral lung metastases** Unknown	36 (14.3) 10 (0.0)	26 (30.5) 7 (8.5)	62 (30.0) 17 (8.2)	.757
**Brain metastases** Unknown	12 (14.3) 17 (0.0)	8 (9.5) 10 (13.5)	20 (9.7) 27 (13.0)	.812
**Bone metastases** Unknown	23 (42.9) 15 (0.0)	21 (20.5) 8 (11.5)	44 (21.3) 23 (11.1)	.728
Pleural Metastases** Unknown	88 (28.6) 6 (0.0)	70 (78.0) 3 (4.5)	158 (76.3) 9 (4.3)	.861

Abbreviations: *SCLC* Small Cell Lung Cancer, *NSCLC* Non-Small Cell Lung Cancer, *BAL* bronchoalveolar lavage

### Treatment characteristics of patients according to lung cancer type

**Fig. 2:**
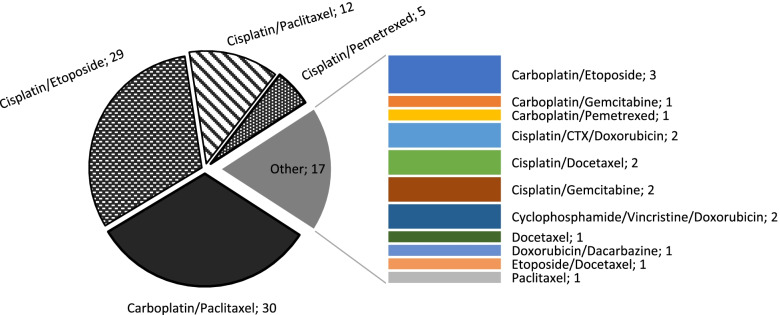
Chemotherapy regimens used for the lung cancer patients

Chemotherapy was the most used modality of treatment and patients below 60 years were likely to be prescribed chemotherapy compared to those above 60 years (54.2% versus 37.6% respectively *p = .024*). Platinum compounds were given to 93.5% (87/93) of patients during their initial cycle of chemotherapy. Forty-seven percent (44/93) of patients received a combination of a platinum compound and a taxane, and the most used chemotherapy regimen was a doublet of carboplatin/paclitaxel (32.3%) followed by cisplatin/etoposide (31.2%). The chemotherapy regimens used for the initial cycle are illustrated in the Fig. 2.

Seventy-one of 72 patients (98.6%) who received biological therapy got Erlotinib. Other drugs given included Gefitinib (3 patients) and Osimertinib (1 patient).

### Survival of lung cancer patients

Of the 207 patients enrolled for the study, 168 (81.2%) were followed up till death or the end of the study (right censored). The vital status could not be ascertained for 39 (18.8%) patients. These were lost to follow up and were censored alive on the date they were last seen. The follow-up range was between 1 day and 55.2 months (median 3.4 months).

**Fig. 3:**
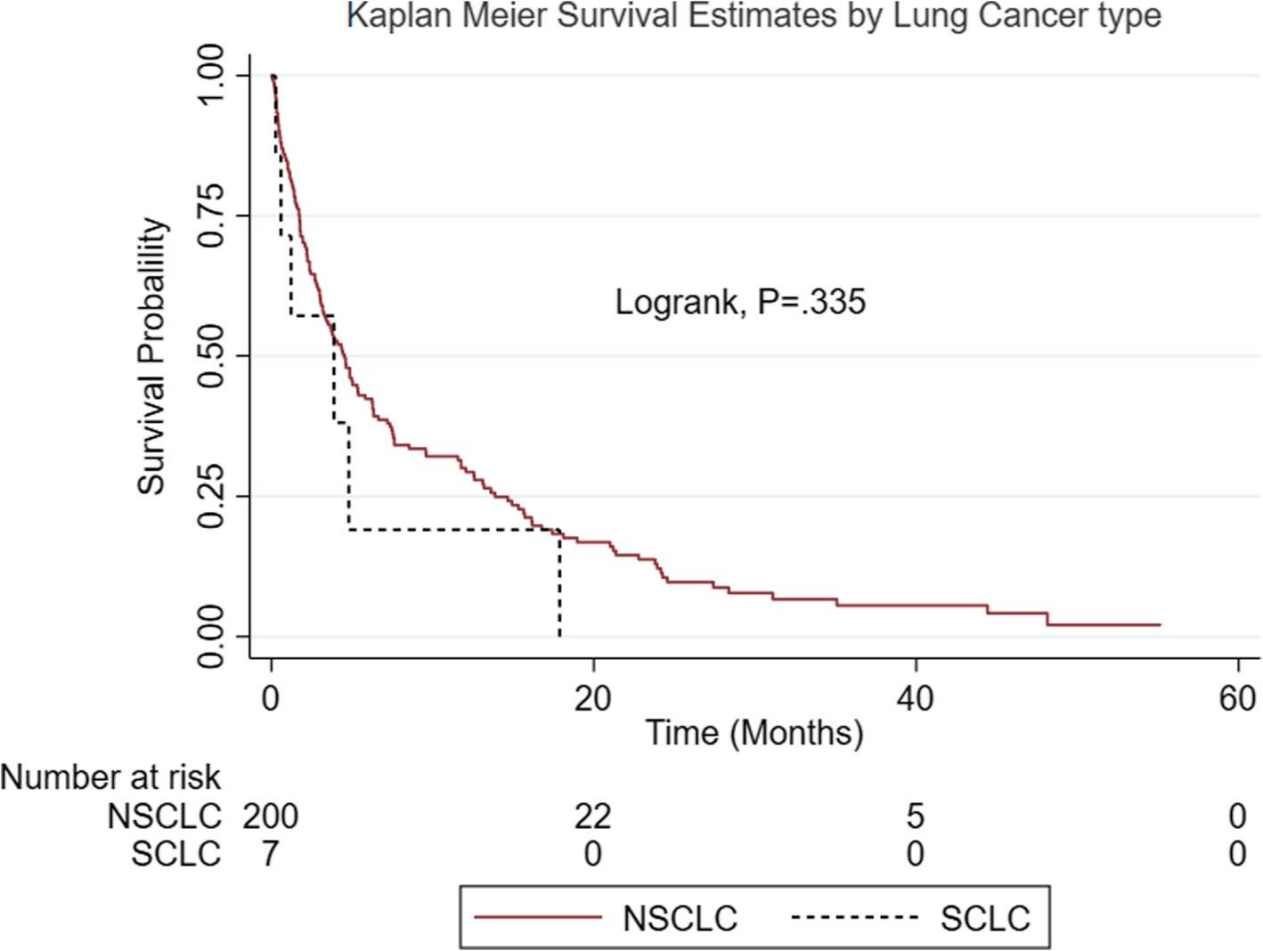
Kaplan Meier survival curves showing 5-year survival of patients according to lung cancer type

The overall, 6-months, 1-, 2- and 5- year survival was 41.7, 29.7, 11.8, and 1.7%, respectively. The median survival time of 4.4 months was not statistically significantly different between NSCLC and SCLC patients (4.5 versus 3.9 months, *p = .335*). The Kaplan Meier survival estimates of patients with NSCLC and SCLC are shown in Fig. 3 below.

**Fig. 4:**
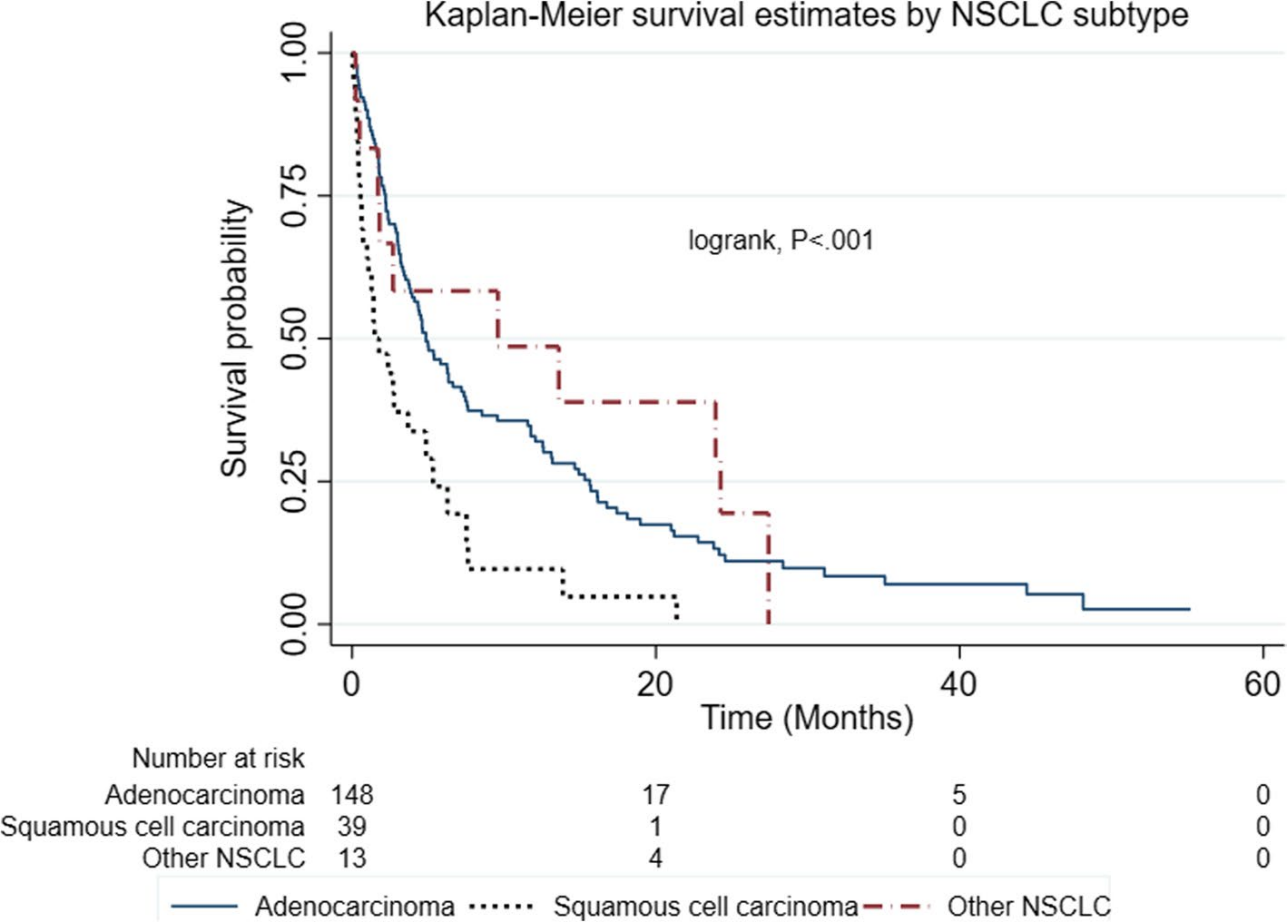
Kaplan Meier survival curves showing 5 years survival of patients according to NSCLC subtype

Within the NSCLC subtype, patients with other NSCLC subtypes combined had better median survival time (9.6 months) followed by Adenocarcinoma patients (4.9 months) and Squamous Cell Carcinoma patients least median survival time (1.8 months); *<.0001*). The Kaplan Meier survival estimates of patients with the adenocarcinoma, squamous cell carcinoma and other NSCLC are shown in Fig. 4 below.

## Discussion

In this study, we set out to describe the baseline characteristics, survival, and prognostic factors among lung cancer patients at Uganda Cancer Institute. The central result of this study was that lung cancer patients at UCI were predominantly female, never-smokers, presented during middle age, with metastatic disease and poor survival. The commonest histological type was adenocarcinoma.

The majority of our patients were middle aged and predominantly female which differed from studies in high-income countries which reported more male patients and median age 10 years older than our patients [6, 7]. This was likely due to the different risk factor profile of our patients with: a lower life expectancy (63.7 years), a lower prevalence (21.8%) of smoking among our lung cancer patients than in the western world (50 to 62%), a high prevalence of HIV (8.7%) among our lung cancer patients, an earlier and longer exposure to biomass fuel among females than males; and a possible increased susceptibility of lung cancer among female patients [8–13].

The symptom profile was similar to more common respiratory illnesses in Uganda like tuberculosis and pneumonia [14]. This resulted in a misdiagnosis of tuberculosis, a longer time from first symptom to diagnosis of lung cancer, delayed treatment referral for lung cancer and exposure to inappropriate anti-tuberculosis treatment. It also highlighted a diagnostic inertia among health workers towards lung cancer and the challenge of making the diagnosis in a country with a high prevalence of tuberculosis, a well described diagnostic chameleon of lung cancer [15]. These delays in diagnosis and treatment of lung cancer can lead to missed opportunities for both curative and life-prolonging therapies [16].

SCLC was uncommon in our population and was lower than that reported in the Western world (15–20%) where the rates of smoking are high [17–19]. This may be explained by the low smoking rates in our population, yet SCLC is strongly associated with cigarette smoking.

All our patients presented with advanced lung cancer (stage 3 or 4) with 96.1% having stage IV lung cancer. Lung cancer is generally diagnosed at an advanced stage because its symptoms are usually subtle or absent in early disease [20, 21]. The proportion of stage IV lung cancer in our study was higher than those reported in the western world and North Africa which ranged between 47.5 and 82.0% [21–24]. Seventeen (94%) of 18 HIV-positive patients with lung cancer also had stage IV lung cancer at diagnosis despite having been in regular contact with the health system during routine HIV care. This implies that there are health worker-associated factors responsible for the late presentation of our patients as well.

Most lung cancer metastases involved the pleura, the contralateral lung, or bone. Brain metastases were seen in only 9.7% of patients. However, routine imaging to check for bone and brain metastases is not done at UCI due to inadequate resources. Therefore, the proportion of patients with brain and bone metastases may have been understated by our results. NSCLC was prone to involve the pleura compared to SCLC (*P = .004*). This was likely because the majority of our NSCLC patients had adenocarcinoma, which invades the pleura early because of their peripheral location. In addition, certain mutations in adenocarcinomas like the EML4-ALK1 rearrangement make them have a higher propensity for pleura metastasis and malignant effusion [25].

Chemotherapy was the commonest form of treatment followed by biological therapy. This is because chemotherapy and biological therapy have a role in advanced lung cancer while the benefits of surgery and radiotherapy are limited in advanced disease. In line with most international guidelines, platinum-based therapy was the most used first-line chemotherapy at UCI [26]. Drugs that target the EGFR pathway like Erlotinib, Gefitinib and Osimertinib were used for biological therapy despite 91% of the patients who received them having no assessment for EGFR mutation status. This was likely because the laboratory tests to identify patients who may benefit from these treatments are only available in a few private laboratories, which are expensive, and oncologists opt to give the drugs as a trial of therapy without the tests.

The survival of lung cancer in our population was low. The survival was much lower than that reported for patients from the Western world [27]. This pattern however is not surprising because majority (96.1%) of our patients had metastatic disease at presentation which portends a poor survival. Studies done in the western world also showed a 5-year survival rate of less than 3% for patients with stage IV lung cancer [28, 29]. This implies that late stage at diagnosis greatly affects survival of our patients.

Our study was not without limitations. Some information was incompletely documented and this was because such information was either not routinely collected or absent due to omission. Certain information on risk factors like biomass fuel was unavailable for most of the patients. This could have resulted in a confounding bias. The vital status for 18.8% of patients could not be ascertained which introduced an attrition bias. However, there were no significant differences in the baseline characteristics between those lost to follow up and their counterparts.

## Conclusion

Our lung cancer population is middle-aged with a higher proportion of females, never smokers, adenocarcinoma, and advanced stage at diagnosis. Lung cancer presents with symptoms that mimic more common respiratory diseases in Uganda like tuberculosis and it therefore requires a high index of suspicion to make the diagnosis early. There is diagnostic inertia among health workers towards lung cancer and a significant proportion of lung cancer patients are initially misdiagnosed as tuberculosis which delays the diagnosis of lung cancer. The survival of our patients is still very poor and much lower than that in developed countries. The lung cancer prevention and early detection programs promoted by the non-communicable disease department of the Ministry of health need to be tailored to match the profile of patients that we are seeing currently in our population.

## Data Availability

The datasets used and/or analyzed during the current study are available from the corresponding author on reasonable request.
